# Level of physical activity and barriers to exercise in adults with type 2 diabetes

**DOI:** 10.3934/publichealth.2021018

**Published:** 2021-03-09

**Authors:** Carolina Gómez Martin, Maria Laura Pomares, Carolina Maria Muratore, Pablo Javier Avila, Susana Beatriz Apoloni, Martín Rodríguez, Claudio Daniel Gonzalez

**Affiliations:** 1 Cendia (Endocrinology and Diabetes Center), Urquiza 802, EP C, CP 3200, Concordia, Entre Ríos, Argentina; 2 Diabetes Unit CEGYM. San Martin 569, CP 3400. Corrientes, Argentina; 3 Private Office. Pte Kirchner 908. 3ro 10, CP 9400, Rio Gallegos, Santa Cruz, Argentina; 4 Public Employees Insurance (OSEP) Mendoza, Entre Ríos 345, CP 5600. San Rafael, Mendoza, Argentina; 5 Diabetes Service, Austral Universitary Hospital, Av. Juan D Peron 1500, Pilar, Bs As, Argentina; 6 Universitary Hospital, Cuyo National University, CP 5500. Mendoza, Argentina; 7 Department of Famacology, CEMIC University, CP 1431, Buenos Aires, Argentina

**Keywords:** physical activity, exercise, type 2 diabetes, barriers, IPAQ

## Abstract

**Introduction:**

Physical activity (PA) is an important element in type 2 diabetes mellitus (T2DM) management. The aims of this study were to assess the percentage of adults with T2DM who perform PA, according to the intensity level and to describe barriers to exercise and the association between metabolic control and other clinical variables.

**Methods:**

Multicenter, observational, cross-sectional study. Data were collected through the International PA Questionnaire (IPAQ) and the PA Barrier Questionnaire. Adults (18–65 years old) with T2DM from 17 Argentine diabetes centers were included, from May to July 2018.

**Results:**

A total of 270 men (54.9 ± 9.8 years) and 225 women (55.3 ± 9.6 years) were included. Duration of diabetes: 8.2 ± 6.3 years. The BMI in men was 32 ± 10.6 kg/m^2^, whereas that in women was 32.5 ± 7.2 kg/m^2^. The last two HbA1c values were 7.6 ± 1.7% and 7.5 ± 1.6. Results also showed that 12.7% had clinical heart disease, 13.7% had nephropathy, 20.8% had neuropathy, 6.1% had diabetic foot and 14.1% had retinopathy. The level of PA was low in 52.3% of the patients studied and moderate in 30.5%. The most frequent barriers were: “lack of will” (59.6%) and “lack of energy” (37.2%). The low level of PA was associated with age (OR: 1.05 per year of age; p < 0.001), HbA1c (OR: 1.16 per 1%; p < 0.05), BMI (OR: 1.06 per kg/m^2^; p < 0.001) and sex (OR: 1.69 for women; p < 0.01).

**Conclusions:**

PA in a cornerstone in management T2DM. Nevertheless, in this study, 52.3% of T2DM adults showed low level of PA. The main barriers reported were related to low personal motivation. These factors should be taken into account to implement programs to promote physical activity.

## 1. Introduction

Physical activity (PA) plays a key role in Type 2 diabetes mellitus (T2DM) treatment. It has been demonstrated that PA improves metabolic control, decreases the onset and progression of cardiovascular disease, improves symptoms of peripheral neuropathy, and reduces insulin resistance [1]. Previous studies that evaluated the PA level have reported an inverse relationship with death from any cause and cardiovascular events in individuals who perform moderate or high level of PA [2]. Based on this evidence, current guidelines recommend performing at least 150 min of moderate aerobic exercise per week, and two weekly sessions of strength exercises [3],[4].

Based on the IDF Diabetes Atlas 2019, the prevalence of diabetes reported in Central and South America is 9.4% [5]. In Argentina, the Risk Factors National Survey in 2018 showed a rate of high blood glucose or diabetes of 12.7% [6]. Despite the benefits of PA and guidelines recommendations, not all people with diabetes achieves the goals proposed [7]. In the United States of America (USA), 60% of people with diabetes do not achieve the recommendations of 150 minutes of exercise per week [8]. In Argentina a study in general population, showed that 51% performed a low level of PA [9]. This is due to multiple reasons, some of them related to promotion as inadequate doctor's prescription, lack of time in medical visits, and lack of knowledge or resources of diabetes specialists/educators, and others are related to patient's issues [10]. Studies in different countries have also investigated and identified different barriers that people with diabetes face to perform PA [11],[12]. These barriers are related to multiple aspects: socioeconomic, psychosocial, cultural, environmental factors etc., so the barriers patterns vary widely in different backgrounds [7],[13]–[16]. In this way, evidence on level of PA and barriers in sub-groups of populations as people with diabetes in the Latin America is scarce, but is a potential area of concern. A study in 85 adults with T2DM, in a unique center in Venezuela using the International Physical Activity Questionnaire (IPAQ) showed 17.6% of low PA [17] and two studies in Peru, using the same questionnaire, including 120 and 164 persons with diabetes reported 20,7% and 20% respectively [18],[19].

There are no published data that investigate the level of PA or the barriers to perform PA in people with diabetes in this country, in spite of the relevance of the topic and the impact that PA may have on the health of individuals with diabetes. The access to this information could be useful to develop physical activity programs more suited to our specific population and its needs.

Thus, the main aim of this study was to evaluate the percentage of adults with T2DM who perform PA and to describe the level of such activity, including its frequency, intensity and duration. The secondary objective was to determine the main barriers to perform PA and their relationship with metabolic control, in a sample of adults with T2DM from Argentina.

## 2. Materials and methods

### 2.1. Study design

A multicenter, observational, cross-sectional study was conducted. The study included randomly selected adults with T2DM, who attended medical visits in public and private health centers specialized in diabetes in Argentina, with a total of 30 patients from each center. The study was carried out from May 2 to July 25, 2018. The inclusion criteria were: people diagnosed with T2DM (World Health Organization Criteria 1999) aged from 18 to 65, whom were capable of completing questionnaires.

Patients with recent T2DM diagnosis (less than 6 months), pregnant women, and patients with minor or major amputations in lower limbs or motor disabilities were excluded.

Each patient was asked to sign an informed written consent to participate in the study and then given the following two questionnaires: the International Physical Activity Questionnaire (IPAQ) in its short version [20],[21] and the Questionnaire of Barriers to Physical Activity [22],[23] (Appendice 1). They were next given sufficient time to respond the questionnaires in a self-administered way. In addition, demographic, clinical and biochemical data were obtained from the patient's medical history.

To evaluate the percentage of adults with T2DM who perform PA and to describe the level of such activity the results of the IPAQ [20] were analyzed. According to IPAQ, individuals can be classified into three categories; those who perform low PA: the person does not perform any PA or not reach 600 Metabolic Equivalent of task/minute/week (METs-min/week); moderate PA: any combination of walking and/or PA of moderate and/or vigorous intensity, reaching an energy expenditure of at least 600 METs-min/week; and high level of PA: the person performs vigorous PA for at least 3 days, reaching an energy expenditure of 1,500 METs-min/week, or 7 or more days of any combination of walking and/or PA of moderate intensity and/or vigorous PA, reaching an energy expenditure of at least 3,000 METs-min/week. The variable low level of PA (less than 600 METs-min/week) was selected as a dichotomic variable to analyze the association with other factors.

To determine the main barriers to perform PA the Questionnaire of Barriers to Physical Activity [22],[23] was used. Among the barriers, social influences (or social support) refer to social interaction and the influence of the PA performed by people in the nearest social environment and involves enjoying and committing to others (i.e. “to be active with others”). To assess whether diabetes was perceived as a barrier, in the present study we added the following question: “Do you think diabetes is a limitation to perform PA?”

Demographic data collected from medical records. Weight in kilograms and height in meters were recorded with mechanical scales with altimeter. Glycated Hemoglobin A1c (HbA1c) was determined either by High-performance liquid chromatography (HPLC) (in four of the centers) or by immunoturbidimetry (in the remaining centers) and were recorded the two last values perfomed within the previous 3 months and the previous year respectively. The number of daily self-monitoring blood glucose tests was determined by patients recall. Health system in Argentina has three sectors: public, social security and private: 62% of the population is covered by social security or private services and 36% attended the public system centers [24]. The type of health coverage was recorded from medical records.

Complications of diabetes were recorded from medical record according to the following definitions:

1. Clinical heart disease: a history of myocardial infarction, percutaneous coronary angioplasty and/or heart failure (2013 Association College of Cardiology Foundation/American Heart Association) Guideline for the Management of Heart Failure).

2. Stroke: history of ischemic and/or hemorrhagic cerebrovascular event.

3. Nephropathy: presence of albuminuria greater than 30 mg/24 h, urine albumin-to-creatinine ratio greater than 30 mg/g, and/or reduction of the estimated glomerular filtration rate lower than 60 ml/min in the absence of signs or symptoms of other primary causes of renal damage (American Diabetes Association (ADA) Standards of Care, 2019).

4. Neuropathy: presence of symptoms or signs of peripheral nerve dysfunction in people with diabetes after other possible causes have been excluded (ADA Standards of Care, 2019).

5. Diabetic foot: foot ulcer (Wagner grade 1 to 3).

6. Retinopathy: diagnosed by dilated fundus exam, photocoagulation history and/or vitrectomy.

### 2.2. Statistical analysis

Continuous variables were described through the use of the arithmetic mean and its standard deviation or as the median and its interquartile range, as appropriate to the nature of the distribution. Qualitative variables are presented as frequencies and percentages. Differences between groups of quantitative data were assessed by the Student's t test or the Mann-Whitney test (depending on the nature of the distribution of the quantitative variable), or through one-way ANOVA (Scheffe post-hoc test) or the Kruskal-Wallis test in the case of more than two data groups. The categorical variables were analyzed using the Chi-square test. The univariate association between two variables was explored through Pearson's simple linear regression method or Spearman's non-parametric test, according to the nature of the variable distribution. Univariate logistic regression was also used where applicable. For the multivariate analysis, we used the Multiple Logistic Regression (Maximum Likelihood; Quasi Newton) and Multiple Linear Regression methods. Bilateral tests were used at a significance level of 0.05. The data were recorded in Excel spreadsheets (Microsoft Windows). Prevalences were estimated and are shown together with their 95% confidence intervals (CI). The data were analyzed with the SPSS 16000 analytical software: SPSS Inc. Released 2009. PASW Statistics for Windows, Version 18.0. Chicago: SPSS Inc.

### 2.3. Calculation of sample size

Assuming that about two-thirds of patients with T2DM perform low PA, with an alpha error of 5%, a power of 20%, and an absolute accuracy of 5%, we obtained a sample size of about 450 individuals in conditions to enter the study and provide the necessary information for further analysis of the results.

### 2.4. Ethical considerations

The study was conducted in accordance with the ethical principles for human research in the Declaration of Helsinki 2013 [25] and followed the approval regulations of independent Ethics Committees of the Health Centers where the study was performed. In addition, as mentioned above, each patient signed an informed consent to participate.

## 3. Results

Five hundred four patients were selected to participate in the study. Relevant information on any of the variables was missed in 9 cases. These subjects were excluded from this analysis. A total of 495 people with T2DM, from 17 public and private centers specialized in diabetes from 10 Argentine provinces were included in the study. The duration of diabetes was 8.2 + 6.3 years; 54,9% were men and 58.6% showed a BMI greater than or equal to 30. The main characteristics of the participants are shown in Table 1.

**Table 1.** Characteristics of the participants.

Characteristics of the participants	n = 495
Men n, (%)	270 (54.5)
Age (mean (years) ± SD)	
Men	54.9 ± 9.8
Women	55.3 ± 9.6
T2DM duration(years) (mean ± SD)	8.2 ± 6.3
BMI equal to or greater than 30 n, (%)	290 (58.6)
Average BMI (kg/m^2^) (mean ± SD)	
Men	32.0 ± 10.6
Women	32.5 ± 7.2
HbA1c (last two values in %) Mean ± SD	7.6 ± 1.7
	7.5 ± 1.6
Chronic complications n, (%)	
1. Clinical heart disease	63 (12.7)
2. Nephropathy	68 (13.7)
3. Neuropathy	103 (20.8)
4. Diabetic foot	30 (6.1)
5. Retinopathy	70 (14.1)
Pharmacological treatment n, (%)	
One or more oral agents or GLP1 agonists	309 (62.4)
Insulin (with or without oral agents)	161(32.5)
Health Insurance n, (%)	432 (87.2)

According to the IPAQ results, the level of PA was low in 52.3% of the patients included and moderate in 30.5% of them.

**Figure 1.**
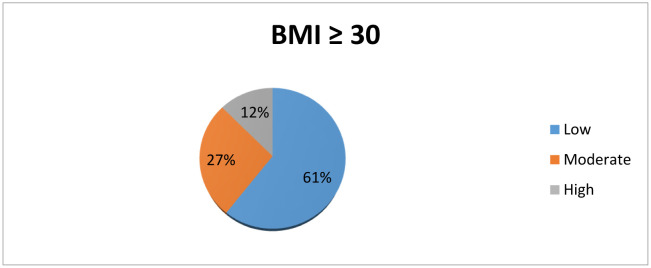
Level of PA. Subanalysis in patients with BMI ≥30.

About 60.6% of the participants with a BMI greater than or equal to 30 recorded low PA, 26.9% moderate PA, and 12.5% high PA (Figure 1).

In the univariate analysis, the low levels of PA were significantly associated with: female sex (r = 0.10; p = 0.0254), age (r = 0.15; p = 0.0007), duration of diabetes (r = 0.11; p = 0.0110), HbA1c values (average of the last two values: r = 0.16; p = 0.0005), BMI (r = 0.13; p = 0.0052), positive history of clinical heart disease (r = 0.12; p = 0.0117), antecedent of stroke (r = 0.03; p = 0.0418), antecedent of nephropathy (r = 0.10; p = 0.0332), history of neuropathy (r = 0.14; p = 0.0023), history of diabetic foot (r = 0.14; p = 0.0020), retinopathy (r = 0.11; p = 0.0188), number of daily self-monitoring blood glucose tests (r = 0.10; p = 0.0301), lack of social support (r = 0.19; p < 0.0001) and current treatment (r = 0.13; p = 0.0037).

In the multivariate logistic regression analysis, the low PA levels determined by the IPAQ were associated with age (OR: 1.05 per year of age; p < 0.0010), HbA1c values (OR: 1.16 per 1%; p < 0.0489), BMI (OR: 1.06 per 1 point in kg/m^2^; p < 0.0010), diabetic foot (OR: 2.77; p = 0.0511), sex (OR: 1.69 for women; p < 0.0120) and lack of social support (OR: 2.42).

**Table 2.** Variables associated with low PA levels.

	Odds ratio	95% CI
HbA1c (mean)	1.16	1.00–1.34
Age	1.05	1.03–1.08
BMI	1.05	1.01–1.09
Social influences	2.42	1.46–4.00
Diabetic foot	2.77	1.00–8.10
Female sex	1.69	1.06–2.37

**Figure 2.**
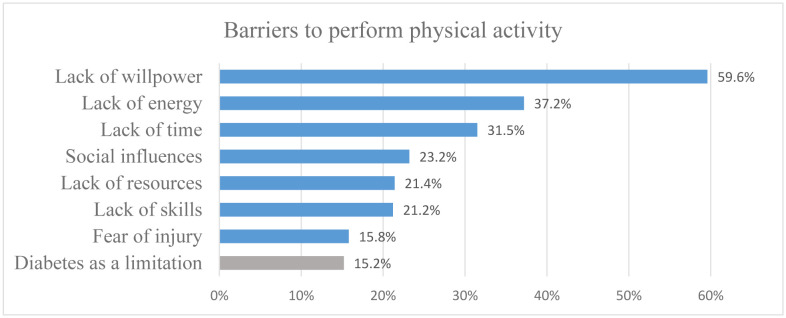
Barriers to perform physical activity. In blue, the variables measured using the questionnaire “Barriers to being active”. In grey, a variable added by the authors: “Do you think diabetes is a limitation to perform PA?”

The results of the questionnaire on barriers to perform PA are shown in Figure 2.

## 4. Discussion

The present study is the first to specifically assess the level of PA in adults with T2DM in Argentina. Results showed that 52.3% of the patients evaluated perform a low level of PA. This low level of PA correlates with older age, higher HbA1c values, diabetic foot history and female sex. Regarding the barriers to perform PA, the ones most reported were lack of willpower, lack of energy and lack of time. Social influences has been a barrier in one out of four participants. In contrast, diabetes as a barrier was the least mentioned.

A previous study performed in Argentina in 2015, in general population, found low PA in 51% of the people evaluated [9], a value similar to that recorded in the present study. In studies performed in other countries of South America, using the same questionnaire (IPAQ), the PA level recorded in adults with T2DM was lower than that recorded in our study: 17.6% of low PA in Venezuela [17] and 20% in Peru [18],[19]. In a systematic review that included 29 studies from 15 countries, the (IPAQ) was the most common resource (11 studies) and four studies used researcher-developed questionnaires, high variability was shown: 15% to 61% of the people evaluated reached the current target of 150 minutes per week of moderate to vigorous PA [26]. The diversity of methods to evaluate the PA level and the different metrics with which the results are reported makes it difficult to compare between the different studies. Even studies using the IPAQ have shown heterogeneous results [17]–[19]. This may be because questionnaires appeal to the participant's memory and tend to overestimate PA in domestic and work activities [27].

According to the factors associated to low PA levels, body mass index (BMI), age and female sex, in our data are consistent with most previous studies. BMI and age were inversely correlated with PA level [10],[12] and women had higher levels of low PA [12]. Obesity was found to be a barrier to being active, due to the physical limitations and habits associated with excess weight [12]. In relation to metabolic control, our results showed that the low level of PA was significantly correlated with higher HbA1c values. These results are similar to those obtained by Palermo et al. in the Philippines [28]. However, another study performed in Oman reported no differences in the metabolic control of physically active patients [14].

With regard to the barriers to perform PA, lack of willpower which refers to a difficulty to control one's own actions, emotions or lack of determination; lack of energy which refers to feeling tired or sleepy; and lack of time, are related to personal motivations. Those factors have also been found to be very common in different studies [7],[15]. Overcoming these barriers to physical activity may require the promotion of self-efficacy, defined as “people's beliefs about their capabilities to exercise control over their own level of functioning and over events that affect their lives.” Examples of motivators to exercise identified among adults include an improved sense of well-being and the desire to serve as a positive example for their children [29].

In relation to other barriers, our results showed that social influences constituted a barrier in one every four patients. Similarly, Alghafri et al. found that one of the most frequent barriers were lack of social support [14], although this study was performed in Oman, a population culturally very different from that of Argentina. In the present study, economic resources were reported as a barrier by 21.4% of the T2DM patients studied, which may be related to the fact that 85% of the patients had health coverage, i.e. they were not very low-income patients. Similarly, in low-income adults with T2DM in the USA, physical difficulties and lack of time were more frequent barriers than environmental or economic resource reasons [17]. On the other hand, the low perception of diabetes as a barrier (only 15%) could be related to the fact that diabetes can be an asymptomatic disease for years and that, in many T2DM patients, the risk of hypoglycemia is usually low. In some studies, participants reported the physical difficulties and the severity of diabetes as barriers, but not the fact of having the disease [12],[16].

The results of different studies are heterogeneous mainly because of the different ways of evaluating the barriers and of the social and cultural factors that condition them. In our study, the main barriers reported (lack of willpower, lack of energy and lack of time) were strongly related to the lack of motivation to perform PA. These types of barriers are frequently reported in other studies in adults with T2DM, but with different patterns in each setting.

In the present study, by including adults with T2DM aged between 18 and 65 years old and excluding people with motor disabilities and/or amputations, the study population was relatively homogeneous. In addition, the study was a multicenter study, which recorded data from different regions of the country where the diabetic education given to each patient in each center is similar but not systematized. Moreover, the two questionnaires used were developed by internationally recognized institutions, are validated in Spanish, and are frequently used in epidemiological studies. It is also important to point out that the metabolic control is evaluated by two determinations of HbA1c in each patient, a fundamental fact in the follow-up of patients with diabetes that has not been considered in other PA studies [12].

As for the limitations of the present study, its cross-sectional design does not allow inferring causality in the associations found. In relation to these associations, some of the odds ratio were significant but indicated a relatively small increased risk (e.g., age, BMI) or had extremely wide confidence intervals (e.g., diabetic foot). Even when the OR are expressed per year in the case of age, or per kg/m^2^ for BMI, their strengths seem to be relatively small. The number of variables potentially associated with PA is huge and many of them may be simply lacking in this analysis. The cross-sectional nature of the study design may also affect the perception about the strength of the association as presented in the multivariate analysis. On the other hand, in the case of diabetic foot for instance, its small prevalence provided wide confidence intervals in the multivariate analysis. As expected, confidence intervals calculations for those complications with the lowest prevalences exhibit the highest imprecision”. Although the sample reached the size proposed in the design, it may not adequately represent the whole population, given its limited number. Another weakness of the design was the absence of a control group, which forced comparisons with data from other studies in adults without T2DM. Another feature that could also be a limitation is that the patients evaluated consulted diabetologists and may thus not represent other groups of people with T2DM, who attend, for example, primary care facilities; and 85% of the patients evaluated had either Private Health Care or Social Security, which implies greater access to health. On the other hand, there are potential biases because the questionnaires used are self-administered, and there may be errors in the patient's perception. This can even lead to discrepancies between studies using the same questionnaire. Finally, in relation to the metabolic evaluation, HbA1c determinations were not centralized, since four of the centers used the HPLC method and the remaining ones used immunoturbidimetry, a fact that could have generated some heterogeneity in the results.

## 5. Conclusions

In the present study, more than 50% of T2DM patients evaluated were physically inactive. The main barriers identified, i.e. lack of willpower, lack of energy and lack of time, were related to low personal motivation. Identifying the levels of PA and the barriers patterns in this specific population is fundamental to implement the best strategies to improve PA level in persons with T2DM to improve the diabetes management. Future studies should evaluate other tools and compare these findings in different populations.
