# Supplementary material for: Level of physical activity and barriers to exercise in adults with type 2 diabetes
Source: AIMS Public Health. 2021 Mar 9;8(2):229–39. doi: 10.3934/publichealth.2021018 (PMC8116187; doi:10.3934/publichealth.2021018)
Supplement: Supplementary file 1 [file publichealth-08-02-018-s001.pdf]

AIMS Public Health

http://www.aimspress.com/journal/aimsph

AIMS Public Health, 8(2): 229–239.
DOI: 10.3934/publichealth.2021018
Received: 07 November 2020
Accepted: 01 March 2021
Published: 09 March 2021

Research article

Level of physical activity and barriers to exercise in adults with type 2 diabetes

Carolina Gómez Martin[1,*], Maria Laura Pomares[2], Carolina Maria Muratore[3], Pablo Javier Avila[4], Susana Beatriz Apoloni[5], Martín Rodríguez[6] and Claudio Daniel Gonzalez[7]

1 Cendia (Endocrinology and Diabetes Center), Urquiza 802, EP C, CP 3200, Concordia, Entre Ríos, Argentina
2 Diabetes Unit CEGYM. San Martin 569, CP 3400. Corrientes, Argentina
3 Private Office. Pte Kirchner 908. 3ro 10, CP 9400, Rio Gallegos, Santa Cruz, Argentina
4 Public Employees Insurance (OSEP) Mendoza, Entre Ríos 345, CP 5600. San Rafael, Mendoza, Argentina
5 Diabetes Service, Austral Universitary Hospital, Av. Juan D Peron 1500, Pilar, Bs As, Argentina
6 Universitary Hospital, Cuyo National University, CP 5500. Mendoza, Argentina
7 Department of Famacology, CEMIC Unversity, CP 1431, Buenos Aires, Argentina

* **Correspondence:** Email: carogamartin@yahoo.com.ar; Tel: +5493454114973.

Appendice 1

1. The International Physical Activity Questionnaire (IPAQ), short version

This questionnaire assesses the PA in the last 7 days in four generic items through seven questions related to work, physical-sports, and transport (walking) activities and to the time the person remains seated or lying down. It is designed to be administered by telephone or self-administered, in young and middle-aged adults (15–69 years), and has been validated both in Spanish [1] and in people with T2DM [2]. Based on the results of the IPAQ, individuals can be classified into three categories: those who perform low, intermediate and high level of PA (Table 1).

Table 1. Physical activity categories determined by the results of the IPAQ [1].

Category	PA level	Activity performed
1	Low	The person does not perform any PA or the one he/she performs is not sufficient to correspond to categories 2 or 3 of the IPAQ.
2	Moderate	3 or more days of vigorous PA for at least 20 min a day, or<br>5 or more days of moderate PA and/or walking at least 30 min a day, or<br>5 or more days of any combination of walking and/or PA of moderate and/or vigorous intensity, reaching an energy expenditure of at least 600 Mets-min/week.
3	High	The person performs vigorous PA for at least 3 days, reaching an energy expenditure of 1,500 Mets-min/week, or<br>7 or more days (sessions) of any combination of walking and/or PA of moderate intensity and/or vigorous PA, reaching an energy expenditure of at least 3,000 Mets-min/week.

2. Questionnaire of barriers to being active

This questionnaire assesses the reasons that prevent a person from being more active. It has been developed by the U.S. Centers for Disease Control (CDC) [3] and validated in Spanish [4]. It explores the reasons for not performing PA, using 21 questions to be answered with one of the following four options: very likely, somewhat likely , somewhat unlikely, and highly unlikely (3, 2, 1 and 0 points respectively), grouped into seven areas or categories (lack of time, social influences, lack of energy, lack of willpower, fear of injury, lack of skills, and lack of resources), with three questions per area. In this questionnaire, a barrier is defined as that when the score of the three questions of each area is equal to or greater than 5. Social influences (or social support) refer to social interaction and the influence of the PA performed by people in the nearest social environment and involves enjoying and committing to others (i.e. “to be active with others”).

References

1. Delgado Fernández M, Tercedor Sánchez P, Manuel Soto Hermoso V (2005) Traducción de La Guía Para El Procesamiento de Datos y Análisis Del Cuestionario Internacional de Actividad Física (IPAQ). *Versiones Corta y Larga*.
2. Caravalí Meza N, Bacardi Gascón M, Armendáriz-Anguiano A, et al. (2016) Validación del Cuestionario de Actividad Física del IPAQ en Adultos Mexicanos con Diabetes Tipo 2. *J Negat No Posit Results* 1: 93–99.
3. Centers for Disease Control and Prevention. HHS, NIH, NIDDK, NDEP. Barriers to being active quiz Accessed July 1, 2020. Available from: http://www.cdc.gov/nccdphp/dnpa/physical/life/barriers_quiz.pdf.
4. Rubio-Henao RF, Correa JE, Ramírez-Vélez R (2015) Propiedades psicométricas de la versión al Español del cuestionario “Barriers to being active quiz”, entre estudiantes universitarios de Colombia. *Nutr Hosp* 31: 1708–1716.

AIMS Press

© 2021 the Author(s), licensee AIMS Press. This is an open access article distributed under the terms of the Creative Commons Attribution License (http://creativecommons.org/licenses/by/4.0)
